# Alpha-1 Antitrypsin Deficiency in Iranian Patients with Chronic Obstructive Pulmonary Disease

**DOI:** 10.5812/ircmj.7508

**Published:** 2013-11-05

**Authors:** Bita Geramizadeh, Zahra Jowkar, Leila Karami, Masoum Masoumpour, Samrad Mehrabi, Mohammad-Ali Ghayoumi

**Affiliations:** 1 Department of Pathology, Transplant Research Center, Shiraz University of Medical Sciences, Shiraz, IR Iran; 2 Transplant Research Center, Shiraz University of Medical Sciences, Shiraz, IR Iran; 3 Department of Internal Medicine, Shiraz University of Medical Sciences, Shiraz, IR Iran

**Keywords:** Alpha-1 Antitrypsin Deficiency, COPD, Iran

## Abstract

**Background:**

Alpha-1 antitrypsin deficiency is a genetic disease which affects both lung and liver. This disease is a recognized factor for chronic obstructive pulmonary disease (COPD). However its importance as the cause of COPD in a country such as Iran is unclear.

**Objectives:**

This study was conducted to find out the role of α-1 antitrypsin deficiency as a cause of COPD in Iranian patients.

**Materials and Methods:**

The serum concentration of α-1 antitrypsin was determined and the genotype of α-1 antitrypsin was also evaluated by PCR-RFLP in 130 patients with COPD and 50 normal healthy blood donors.

**Results:**

No α-1 antitrypsin deficient case was found in normal healthy people and COPD patients.

**Conclusions:**

Our results clarify that deficiency of α-antitrypsin is not a major cause of COPD in Iranian patients.

## 1. Background

Αlpha-1 antitrypsin (AAT) is an acute phase reactant mostly produced by the liver and its major function is to inhibit neutrophil elastase and protect lungs against the enzyme action (1). AAT gene is located on chromosome 14q32. It is a highly polymorphic gene with over 100 alleles identified, at present (2). The SERPINA1 PI*M allele codes for the normal AAT variant, whereas PI*S and PI*Z are the most common deficiency alleles. Deficiency alleles other than PI*S and PI*Z have very rarely been reported (1). AAT deficiency is associated with severe pulmonary emphysema and chronic obstructive pulmonary disease (COPD) (3). The prevalence of AAT in COPD patients varies from one country to another (4). WHO has advised screening for AAT in all COPD patients once in their life (5). To the best of our knowledge there is no published study in the English literature from Iran about the prevalence of AAT deficiency in COPD patients.

## 2. Objectives

We conducted this study to find out the role of this deficiency as a cause of COPD.

## 3. Material and Methods

During a one year period (2009 - 2010), 130 patients who referred to with the symptoms of COPD (cough, dyspnea, wheezing and shortness of breathing) and showed FEV1 < 80%, which is the diagnostic criteria for COPD, as recommended by American society of thoracic diseases were selected for the study (6). For each patient 10 cc of blood (5 cc clot and 5cc in EDTA) was collected.

DNA was isolated from the whole blood (in EDTA) using phenol-chloroform procedure and was frozen at -20°C. PCR-RFLP (Polymerase chain reaction-restriction fragment polymorphism) was performed with the below-mentioned primers. Primes for Z mutation were as below:

PFZ: 5-ATAAGGCTGTGCTGACCATCGTC-3 (T_m_: 65°c)

PRZ: 5-TTGGGTGGGATTCACCACTTTTC-3(T_m_: 63°c)

Primers for S mutation were as below:

PRS: 5-TGAGGGGAAACTACAGCACCTCG-3 (T_m_: 66°c)

PRS: 5-AGGTGTGGGCAGCTTCTTGGTCA-3 (T_m_: 66°c)

The PCR reaction mixture is shown in Table 1. 

**Table 1.** The PCR Mixture

PCR	Volume, tub, µL	Final Concentration
**10 X PCR buffer**	5	1X
**50 mM mgcl**_**2**_	1.5	1.5 mM
**10mM dNTPS**	1	200 µM
**10mM PF&PR **	2.5	0.5 µM
**5 unit / µl polymerase**	0.5	2.5 unit
**Distilled water**	32	-
**Template DNA**	5	-

The PCR temperature and cycles are shown in Table 2. This schedule was the same for S and Z primers. 

**Table 2.** The PCR Temperature and Cycles

Round	Profile	Denaturation	Annealing	Extension	None of Cycles
**First**	Temperature, °C	94	-	-	1
	Time, min	10			
**Second**	Temperature, °C	94	60	72	35
	Time, s	2	2	2	
**Third**	Temperature, °C	-	-	72	1
	Time, min	-	-	10	

To test for Z and S alleles’ mutation, an aliquot of the PCR product (20 µL) was digested with 10 units (taq1) of restriction enzyme and 3µl of 10X buffer (on ice), then distilled water was added to reach final volume of 30 µL. After mixing gently and centrifuging at 3000 rpm for 20 seconds, the sample was incubated at 65°C for 16 hours. Upon digestion, 10 µL of cleaved product was submitted to electrophoresis in 3% agarose gel for one hour and stained by Ethidium Bromide.

**Figure 1.**
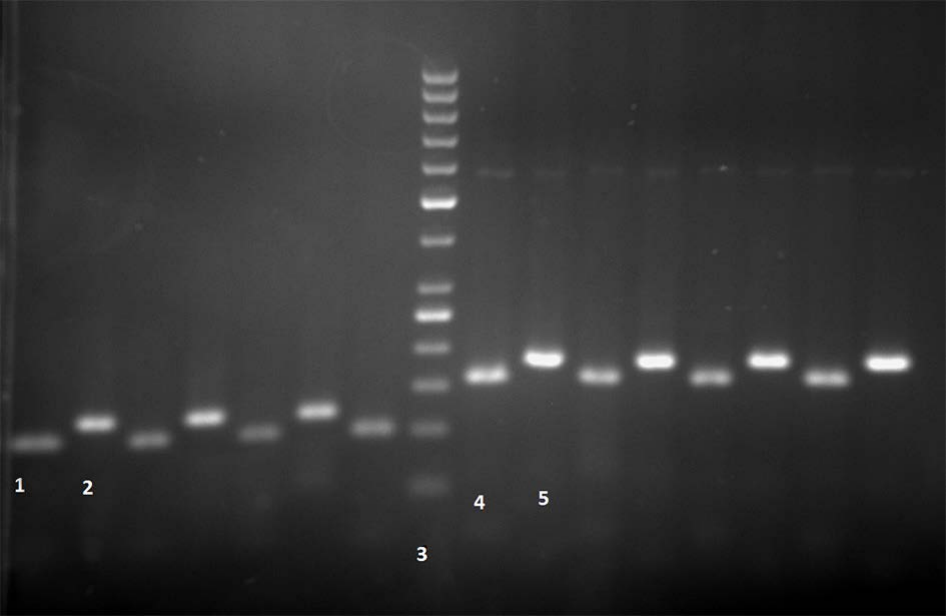
S Mutation Lane 1, MM Genotype: 100bp (after enzyme); Lane 2, PCR product: 121bp (before enzyme); Lane 3, 50bp ladder; Z mutation: Lane 4, MM Genotype: 157bp (after enzyme); Lane 5, PCR product: 179bp (before enzyme)

Normal alleles will be digested by the taq1 enzyme resulting in 157bp and 22bp fragments, but the enzyme will not digest the mutant alleles. Water was used as a negative control (Figure 1). 

In the meantime, the level of AAT was measured in the serum by nephlometry. (Minineph TM human AAT kit, product code ZK034R).

## 4. Results

130 COPD patients were included in this study. The age range was 21 to 70 years (mean = 48.7 ± 8). 126 patients were male and only four females were selected. 125 patients were smokers.

59 healthy blood donors with normal respiratory function were also selected for the control group with mean age of 28.4 years (51 males and 8 females). The AAT level by nephlometry was normal in all patients and controls (0.629 - 1.696 gr/L). All patients and controls showed MM genotypes and no mutant cases were detected. The PCR products were also analyzed by DNA sequencing and this analysis also confirmed the MM genotype. So in these COPD and healthy groups of people, no case of AAT mutation and deficiency was identified.

## 5. Discussion

AAT is a 52 KD glycoprotein mostly secreted by hepatocytes but also secreted by other cells such as lung epithelial cells and phagocytes (7). The specific target of AAT is neutrophil elastase, an enzyme that digests elastin, basement membrane and other extracellular matrix components (8). AAT deficiency causes predisposition to develop a number of diseases through life, mainly COPD and several types of liver diseases (4). PiMM is the normal and most common allele, but AAT gene is highly polymorphic with more than 100 alleles. Variants are classified according to the protease inhibitor (Pi) system (9). Most of the pathology is related to the Z allele and more than 96% of the patients worldwide have a ZZ phenotype and the remaining 4% mostly belong to the SZ and MZ variants (4).

Prevalence of AAT deficiency varies from one country to another and knowledge about the AAT deficiency status in every country is essential (10). It is estimated that 34 million people worldwide are deficient for AAT (PiZZ, PiSZ, PiSS) and 116 million are carriers (11).

AAT deficiency is mostly prevalent in European countries with high frequencies in Spain and Portugal (11). There are very few studies on AAT deficiency as a cause of COPD from Asian countries, most of which have concluded that AAT deficiency is a rare cause of COPD in these countries (1, 8, 12-14). In Hong Kong, a study on 356 COPD patients with mean age of 72.4 years old and 185 healthy unrelated controls identified 63% homozygote for M type and 4% heterozygote for M type and none were homozygous for any deficient allele (14). Based on a study on Korean patients with COPD, involving 114 patients and 196 healthy controls, same distribution of variants in both groups was identified, but S and Z alleles were not found (11). According to another study in Korea on 56 male emphysematous patients above 50 years old serum level of AAT was assayed, also phenotyping and genotyping were done; no S or Z variant was identified and M variant among COPD patients and healthy group was similar (12). In Saudi Arabia, in 158 healthy individuals, genotyping was done which showed 2.53% were heterozygous for Z mutation, 11.39% were heterozygous for S mutation and 3.8% for SZ. Homozygous for SS was present in 1.9% of individuals and no ZZ phenotype was detected (8). In China, serum analysis and isoelectric focusing electrophoresis of 748 normal individuals and 414 COPD patients did not find PiZ or PiS mutations in both groups and suggested that AAT deficiency is not a factor in the development of COPD (13). In Turkey, investigation on 123 healthy blood donors by isoelectric focusing and nephlometry determined no Z and S homozygous variant (15). In India, genotyping by PCR-RFLP technique on 200 COPD patients showed only 2 SS allele (1%) and ZZ variant (0.5%) (16). Based on these studies and current the study in Iran, it seems that AAT is not a common cause of COPD development in the Asian population. However, there is no published study in the English literature about the frequency of AAT deficiency among COPD patients from Iran. According to our study AAT deficiency is definitely not a major cause of COPD in Iran which is the same as other Asian countries.
